# Prediction Value of Serum NGAL in the Diagnosis and Prognosis of Experimental Acute and Chronic Kidney Injuries

**DOI:** 10.3390/biom10070981

**Published:** 2020-06-30

**Authors:** Weida Wang, Zhaojun Li, Yuanyuan Chen, Haijie Wu, Sen Zhang, Xiaoguang Chen

**Affiliations:** State Key Laboratory of Bioactive Substances and Functions of Natural Medicines, Institute of Materia Medica, Chinese Academy of Medical Sciences & Peking Union Medical College, Beijing 100050, China; wangweida@imm.ac.cn (W.W.); lizhaojun@imm.ac.cn (Z.L.); 18581471108@163.com (Y.C.); attitudewu97@163.com (H.W.)

**Keywords:** serum NGAL, experimental kidney disease, ROC curve, biomarker, early diagnosis, prognosis

## Abstract

Sensitive and accurate serum biomarkers for monitoring acute and chronic kidney disease progression are more convenient and can better evaluate drug efficiency in pharmacological research. Neutrophil Gelatinase-associated Lipocalin (NGAL) is considered a hopeful early biomarker of acute kidney injury (AKI), but its utility in early prediction and prognosis of diabetic nephropathy (DN) and immune-mediated glomerulonephritis is still not clear. Moreover, detailed prognosis studies of NGAL in AKI are lacking, and most studies use a urine source. In the current study, through two experimental AKI and two chronic kidney injury animal models, serum NGAL (sNGAL) prediction values on diagnosis and prognosis of kidney injuries in animal disease models have been investigated thoroughly. Four experimental kidney disease models include cisplatin-induced and lipopolysaccharide (LPS)-induced AKI, streptozocin-induced diabetic nephropathy (DN), and cationized-bovine serum albumin (c-BSA)-induced membranous nephropathy (MN), respectively. The sNGAL concentration was measured at different stages of kidney injury (KI) in each experimental model, and receiver operating characteristic (ROC) analyses were performed to investigate the diagnosis efficiency of sNGAL for KI. Western blot and immunohistochemistry were used to measure the protein levels in the kidneys, and pathological analysis was used as the gold standard to confirm KI. Results suggest that sNGAL can predict early diagnosis of cisplatin-induced AKI accurately but is less powerful in later stages compared to blood urea nitrogen (BUN) and serum creatinine (Scr). sNGAL is sensitive but lacks specificity to evaluate early kidney injury for LPS-induced AKI under low-dosage LPS challenge. sNGAL is not an efficient biomarker for early diagnosis of STZ-induced DN, but sNGAL is an efficient predictor for the early diagnosis and prognosis of immune-mediated MN. In conclusion, application of sNGAL as a kidney injury biomarker to determine the diagnosis and prognosis in pharmacological studies is dependent on experimental animal models.

## 1. Introduction

Experimental kidney injury (KI) animal models are very helpful in screening potential nephrotoxic reagents and possible anti-KI compounds in vivo, and they also can provide research tools to study renal pathophysiology. However, traditional biomarkers measuring renal functions in the human study, such as blood urea nitrogen (BUN) and serum creatinine (Scr), are not sensitive enough to detect early or slight damages in experimental animals [1,2]. Urine-derived biomarkers such as albumin, vanin-1, N-acetyl-β-d-glucosamininidase, and kidney injury molecule-1 might be effective biomarkers [3,4]; however, in some conditions, especially when a large number of animals are used in the same experiment batch, serum-derived KI biomarkers might be more advantageous becaue of their shorter identification time and simple extraction. In addition, for acute kidney injury (AKI) animals, it is more difficult to obtain enough urine because serious anuria generally occurs during AKI [5]. Therefore, searching for sensitive, specific, and accurate biomarkers from serum is valuable for experimental KI animals. 

Neutrophil gelatinase-associated lipocalin (NGAL), also called lipocalin 2, is a small secreted glycoprotein of 25 kDa mainly expressed by neutrophils but also by many other cell types including renal tubular cells and podocytes [6,7,8]. Several roles have been ascribed to NGAL, including iron trafficking and chemotactic and bacteriostatic effects [8]. Current knowledge suggests that NGAL is a promising KI biomarker, especially in the early stages of AKI [2]. For example, NGAL is one of the most highly induced proteins in the kidney after ischemic or nephrotoxic AKI, both in clinical studies and animal models, and it increases in urine before the rise in serum creatinine concentration [9,10]. However, most research with experimental animal models lack in-depth predictions of serum NGAL (sNGAL). Additionally, there are few studies about the predictive capability of sNGAL in experimental chronic kidney disease, such as diabetic nephropathy (DN) and immune-mediated glomerulonephritis. Most researchers use human samples and mainly investigate urinary NGAL (uNGAL), and there is also a lack of careful comparisons with BUN and Scr. The structure between human NGAL and mouse or rat NGAL has little homology (62% and 63% respectively) [8,11]. Several biofunctions in humans cannot be detected in rodent animals (e.g., NGAL/MMP-9 interaction is absent in rodents), and rodents are unable to form homodimers and heterodimers [12]. These discrepancies limit the applicability of rodent models in discovering human biofunctions [13,14]. Therefore, a comprehensive investigation of sNGAL in experimental KI animal models will benefit its usage as a biomarker in pharmacology and toxicity studies.

In the current study, four widely used experimental KI animal models, including DN, immune-mediated glomerulonephritis, and two AKI models have been established, and the dynamic levels of sNGAL during disease progression were recorded. The predictive capability of sNGAL, instead of uNGAL, in different phases of KI has been investigated.

## 2. Materials and Methods

### 2.1. Animals and Ethics Declaration

All animals in the current study, including mice and rats, were purchased from SPF Biotechnology Ltd. (Beijing, China). They were housed in a specific pathogen-free facility with free access to food and water, under a constant temperature (22 ± 2 °C) and a daily illumination period of 12 h (7:00 a.m. to 7:00 p.m.).

This study was carried out in accordance with recommendations of the “Chinese guidelines for Proper Conduct of Animal Experiments, Ethics Committee of Laboratory Animals of Beijing Municipality”. The protocol was approved by the “Ethics Committee of Laboratory Animals of Chinese Academy of Medical Sciences”. The approval number was 780875. At the end of experiments, all animas were euthanized by cervical dislocation.

### 2.2. Establishment of Acute and Chornic Kidney Injury Models

#### 2.2.1. Establishment of Cisplatin-Induced Acute Kidney Injury Model

**Figure 1:**
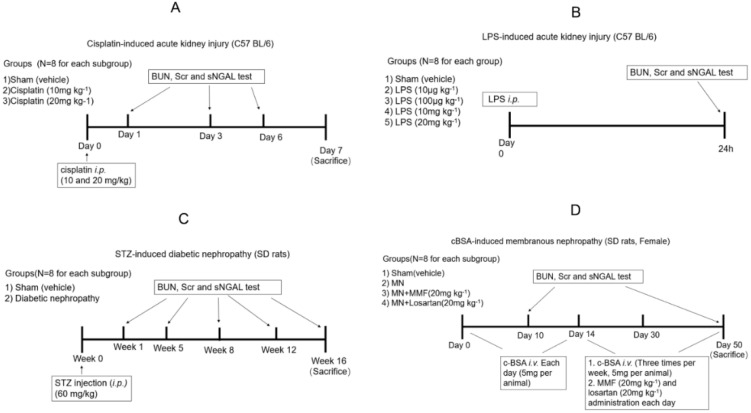
Experimental scheme of four acute kidney injury (AKI) and chronic KI (CKI) animal models. (**A**) Cisplatin-induced AKI; (**B**) LPS-induced AKI; (**C**) STZ-induced diabetic nephropathy; (**D**) c-BSA induced membranous nephropathy.

Adult male C57BL/6 mice (18–20 g) aged 6–8 weeks were used in this study. All mice were divided randomly into three groups as follows: sham control group (NC; *n* = 24) received intraperitoneal (i.p.) injection of vehicle solution (0.9% saline; 10 mL kg^−1^), and the second and third groups were cisplatin AKI groups (*n* = 24) that received a single i.p. injection of 10 or 20 mg kg^−1^ cisplatin, respectively, dissolved in 0.9% saline. An illustration of the animal experimental procedure is shown in Figure 1A. The cisplatin injection day was considered as day 0.

Sham and AKI rats from each group were divided into three subgroups to collect blood samples and kidney tissues at three time points (*n* = 8 for each subgroup). At day 1, day 3, and day 6 blood was collected from the eye endocanthion using a capillary glass tube. Then, animals were euthanized, and kidneys were collected for pathological analysis. The sera samples were collected from blood by centrifugation at 1500× *g* at 4 °C for 15 min for BUN, Scr, and NGAL examinations.

#### 2.2.2. Establishment of LPS-Induced Acute Kidney Injury Model

Forty adult male C57BL/6 mice (18–20 g) aged 6–8 weeks were used in this study. Experimental septic AKI was established by LPS injection (i.p., 10 μg kg^−1^, 100 μg kg^−1^, 10 mg kg^−1^, and 20 mg kg^−1^, respectively; *n* = 8). Sham controls received the same volume of vehicle intraperitoneal injection (*n* = 8). Twenty-four hours later, the blood was collected from the eyes, and serum was harvested for BUN, Scr, and NGAL tests. Then, all animals were euthanized, and kidneys were collected for pathological analysis. A detailed procedure is shown in Figure 1B.

#### 2.2.3. Establishment of STZ-Induced Diabetic Nephropathy

Adult male Sprague-Dawley (SD) rats, 10 weeks old, weighing 180–200 g, were used to induce diabetic nephropathy. Animals were randomly allocated into sham control or diabetic groups (*n* = 40 for each group). Animals were subjected to experimental induction of progressive diabetic nephropathy by injection of streptozocin (60 mg kg^−1^) after overnight fasting, which was diluted in citrate buffer solution (0.1 mol L^−1^ citric acid and 0.1 mol L^−1^ sodium citrate, pH 4.5); the sham group received intraperitoneal injection of the same volume of vehicle. Rats with blood glucose concentrations more than 20 mmol L^−1^ four days after induction of diabetes were included in the study. Diabetic rats were again divided into five subgroups to collect blood sample and kidney tissues at different time points (*n* = 8 for each subgroup). At 1, 5, 8, 12, and 16 weeks after STZ injection, blood was collected to measure BUN, SCr, and NGAL. Animals from subgroups were euthanized, and kidneys were collected for pathological analysis. A detailed experimental scheme is shown in Figure 1C.

#### 2.2.4. Establishment of Cationized-BSA (c-BSA)-Induced Membranous Nephropathy

Female SD rats, 10 weeks old and weighing 160–180 g, were used in the present study. C-BSA induction of membranous nephropathy (MN) was adopted with previous methods [15,16], and the experimental scheme and grouping are shown in Figure 1D. Briefly, 50 rats received intravenous injection of C-BSA (5 mg per animal, dissolved in 0.5 mL sterile 0.01 M PBS, pH 7.4) through the tail vein every day for 14 days, and the first day of receiving c-BSA was considered as day 1. Albuminuria was determined by an albumin ELISA kit (Abcam plc, Cambridge, MA, USA). All established MN rats were randomly divided into the model group (*n* = 24), MMF-treated group (*n* = 8), and losartan-treated group (*n* = 8). MMF (20 mg kg^−1^) and losartan (20 mg kg^−1^) were orally given daily to evaluate whether sNGAL could predict the treatment effect and prognosis of c-BSA-induced MN. Another 24 animals were used as sham control and received PBS i.v. injection following the same protocol. Animals from sham and MN model groups were randomly divided into three subgroups to collect blood and kidney tissues (*n* = 8 for each subgroup). At day 10, day 30, and day 50 after c-BSA i.v. injection, blood and kidney tissues from sham and MN subgroups were obtained. The animals in MMF-treated and losartan-treated groups were euthanized at day 50 to obtain blood and kidney tissues.

### 2.3. Cell Culture

Human kidney-2 (HK-2) cells, an immortalized human proximal tubular cell line, were purchased from American Type Culture Collection (Mannassas, VA, USA). The cells were grown in DMEM medium (Invitrogen, Paisley, UK) supplemented with 10% Fetal Bovine Serum (FBS, Hyclone, Australia), and passage numbers were less than 10. The cell lines were complemented with 2 mmol/L glutamine, 100 U/mL penicillin, and 100 U/mL streptomycin and maintained in a 37 °C, 5% CO2 humidified atmosphere. Cultures were re-fed with fresh media every 2 to 3 days. Cells were incubated with various concentrations of LPS (100, 50, 25, 12.5 μg/mL) for 24 h, respectively, and cells were sent to total protein isolation.

### 2.4. Western Blot

To determine the protein level of NGAL in kidney tissues or HK2 cells, renal cortical tissues or cells were homogenized using ice-cold RIPA lysis buffer (Applygen Biotechnology, Beijing, China) and quantified by the bicinchoninic acid assay (Applygen Biotechnology) with BSA as standard. Equal amounts of protein were separated by sodium dodecyl sulphate-polyacrylamide gel electrophoresis and electroblotted onto polyvinylidene difluoride membrane. After blocking with 5% (*w*/*v*) skim milk solution for 1 h, the membranes were incubated with anti-NGAL antibody 1:1000 (Abcam plc, Cambridge, MA, USA) overnight. β-Actin (Santa Cruz Biotechnology, San Diego, CA, USA) was used as the internal standard. The membranes were then incubated with horseradish peroxidase-conjugated secondary antibody immunoglobulin G (IgG) at 1:2000 dilution for 1 h at 24–28 °C after being washed thrice using Tris-buffered saline with 0.1% Tween 20 (TBS/T). The immunoreactive bands were visualized using an enhanced chemiluminescence system (LAS 4000; GE healthcare, CA, USA). The intensity of the detected bands was analyzed using the Image J program (Version 1.38). 

### 2.5. Biochemical Test and NGAL ELISA Assay

BUN and Scr levels were measured using commercial kits (Beijing Beihua biotechnology, Beijing, China). Serum NGAL levels from mice and rats were measured using a commercially available ELISA kit (Abcam). Urine kidney injury molecule-1 (KIM-1) and urine clusterin-1 from mice were measured by commercially available ELISA kits from Abcam.

### 2.6. Histology and Immunohistochemistry

The bilateral kidneys from all animals were fixed in 10% phosphate-buffered formalin solution and embedded in paraffin. Sections 2 μm in thickness were cut and stained with haematoxylin and eosin (H&E) or Periodic acid-Schiff (PAS) to assess alterations in glomerular (capillary and mesangial) and proximal convoluted tubules. The sections were panoramically scanned, and the images were digitized onto a computer screen using a charge-coupled device camera (S610; Hamamatsu Photonics, Tokyo, Japan) linked to the image analysis software (NDP Viewer 2; Hamamatsu Photonics, Tokyo, Japan). Two independent, experienced pathologists performed histopathological examination in a blinded manner. For each animal, at least 10 high-power (×200) fields were examined. The histopathological injuries in the current study were scored by the percentage of tubules that displayed cellular necrosis, loss of brush border, cast formation, vacuolization, and tubule dilation as follows: 0 (none), 1 (<10%), 2 (11–25%), 3 (26–45%), 4 (46–75%), and 5 (>76%). Grade 0 and 1 were considered as no injury, and grades 2–5 were considered as injury. We used this binary (yes/no) method so we could analyze the outcome using a receiver operating characteristic (ROC) curve. Although glomerular injury was also observed in DN- and c-BSA-induced MN models, only tubular injury was accounted for in the current study because renal NGALs are mostly produced by impaired tubular cells.

Aiming to understand the expression and distribution of NGAL in whole-kidney tissue sections, immunohistochemical staining was performed as previously reported [16,17]. Briefly, kidney tissue sections were dewaxed with xylene and hydrated with ethanol at different concentrations. After that, sections were subjected to microwave antigen retrieval, and endogenous peroxidase was blocked with hydrogen peroxide. Sections were incubated for 45 min at 37 °C with primary antibodies against NGAL (1:200; Abcam). In negative controls, the primary antibody was replaced by buffer. After primary antibody incubation, sections were incubated with a peroxidase-conjugated secondary antibody (ZSGB-Bio, Inc., Beijing, China). Tissues were examined for positive (yellowish-brown) staining and panoramically scanned as mentioned above. Staining density was calculated quantitatively in terms staining distribution and optical density using Image Pro plus 5.0 (Media Cybernetics, Inc., Rockville, MD, USA) based on a five-point grading system.

### 2.7. Statistical Analysis

All data were represented as means ± standard deviation (SD) and analyzed using SPSS statistical software (SPSS Statistical package 24.0, IBM, Chicago, IL, USA). Two-way, repeated measures ANOVA was performed to compare kidney injury biomarkers between each group. The ROC curve was drawn and analyzed by Graphpad prism 8.0 (GraphPad Software, Inc, La Jolla, CA, USA). The outcome predicted by the ROC curve was kidney pathological injury and was defined and confirmed by microscopic observation using tissue sections. *P*-values of less than 0.05 were considered statistically significant.

## 3. Results

### 3.1. Serum NGAL Can Predict Early Injury and Mortality in Cisplatin-AKI but Is Not Advantageous at Later Stages

**Figure 2:**
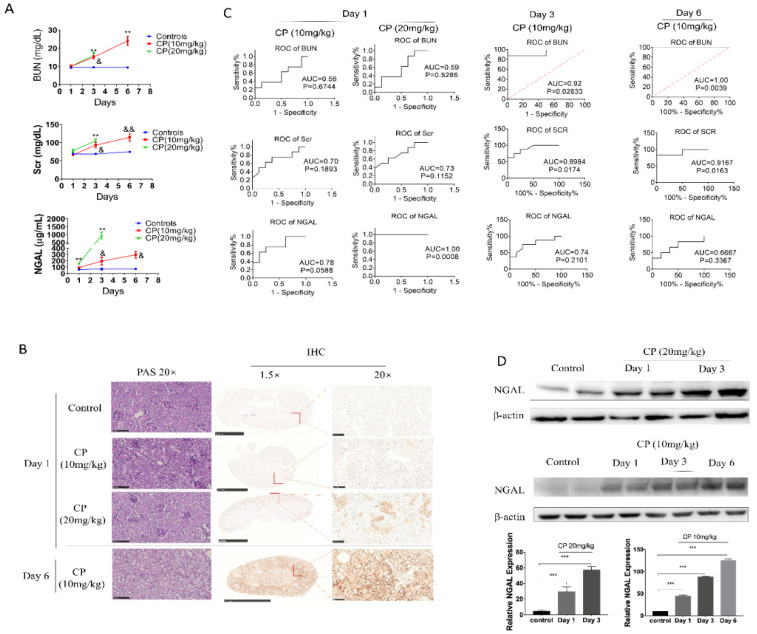
Serum Neutrophil Gelatinase-associated Lipocalin (NGAL) has a superior predictive capability for early kidney injury compared to blood urea nitrogen (BUN) and serum creatinine (Scr) in cisplatin-induced AKI but is not superior at later phases. (**A**) Concentrations of sNGAL, BUN, and Scr in AKI animals at 1, 3, and 6 days after cisplatin injection. (**B**) Representative pictures of Periodic acid-Schiff (PAS) staining and NGAL immunohistochemistry in kidney tissues. (**C**) receiver operating characteristic (ROC) curve comparison of sNGAL, BUN, and Scr to predict AKI at day 1, day 3, and day 6. (**D**) NGAL protein levels increased in kidney tissues in a dose-dependent and time-dependent manner according western blot. CP refers to cisplatin; *, *p* < 0.05, **, *p* < 0.01, ***, *p* < 0.001, 20 mg/kg CP group vs. control; ^&^, *p* < 0.05; ^&&^, *p* < 0.01; 10 mg/kg CP group vs. control (*n* = 8 for each subgroup).

sNGAL, BUN, and Scr in each subgroup were examined respectively at day 1, day 3, and day 6, and their dynamic changes are shown in Figure 2A. Because mice in the 20 mg kg^−1^ group began to die at 72 h after cisplatin injection, the data in the 20 mg kg^−1^ group at day 6 were excluded. sNGAL increased 24 h later in the cisplatin-injected mice, both at 10 mg kg^−1^ and 20 mg kg^−1^ cisplatin dosage, but it was more significant at the 20 mg kg^−1^ dosage (*p* < 0.0001). The BUN and Scr counterparts did not increase significantly compared to sham control mice at day 1 (Figure 2A). Slight histopathologic damage of cisplatin-induced AKI was observed 24 h later in a dose-dependent manner, as shown in Figure 2B. Compared to bright and clear, tubular epithelial cells in the vehicle-treated group, the tubular epithelium from the cisplatin-treated kidney was blurred, and the cell membrane had an abundance of small endocytic vacuoles and contained few glycogen particles at day 1. Immunohistochemistry also demonstrated that cisplatin caused a higher expression of NGAL in the renal tubular area in a dose-dependent manner, but NGAL expression in the glomerular area was not observed (Figure 2B). ROC curves in Figure 2C suggest that sNGAL had 100% diagnosis power at 20 mg kg^−1^ dosage, which was better than BUN and Scr regarding the specificity and sensitivity. At 10 mg kg^−1^, the ROC curve of sNGAL was also superior to that of BUN and Scr (*p* = 0.0588, Figure 2C).

Another phenomenon, as shown in Figure 2A, is that the 20 mg kg^−1^ cisplatin groups had, on average, a sNGAL level 165% higher than that of the sham control (324 ± 85.4 vs. 122 ± 23.9 μg mL^−1^, *p* < 0.0001), while the 10 mg/kg cisplatin group was only 51.6% higher in the current study (185 ± 89.5 vs. 122 ± 23.9 μg mL^−1^, *p* = 0.0747). In the 20 mg/kg group, the lowest sNGAL concentration was 201 μg/mL at day 1, and all the mice in this group died in succession within 6 days. In the 10 mg kg^−1^ dosage group, the three animals with the highest NGAL concentration died within 6 days; among these dead mice, the lowest NGAL concentration at day 1 was 178 μg mL^−1^, which was 45% higher than the average NGAL concentration in the sham group.

Despite the advantages of early diagnosis of sNGAL, its prediction capabilities at day 3 and day 6 were challenged compared to BUN and Scr. As shown in Figure 2A, for the 10 mg kg^−1^ cisplatin group, both BUN and Scr significantly increased at day 3 and day 6, and their prediction capabilities (prognosis ROC value) were higher than NGAL due to the large deviation in the sNGAL concentration (Figure 2C). The animals from the 20 mg kg^−1^ group were not included in the statistical analysis due to serious mortality. Using western blot, we demonstrated that NGAL protein levels in the kidney tissues increased in a time-dependent manner (Figure 2D). Immunohistochemistry also showed the same trend as western blot: NGAL was highly expressed in the whole-kidney tissues from animals in the 10 mg kg^−1^ group at day 6, including the cortex and medulla, and the staining density in the tubular area was significantly darker than in glomeruli (Figure 2B). Typical pathological features in the 10 mg kg^−1^ group at day 6 are also shown in Figure 2C, where widespread tubular necrosis and apoptosis can be seen. 

### 3.2. Serum NGAL Is Sensitive but Lacks Specificity as a Biomarker for LPS-Induced AKI

**Figure 3:**
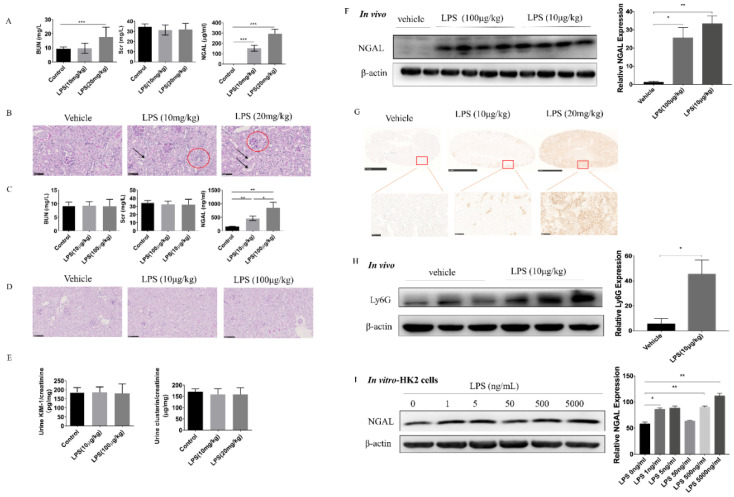
Serum NGAL is a sensitive and accurate biomarker to assess lipopolysaccharide (LPS) -induced kidney injury but is dependent on LPS dosage. (**A**) Concentrations of BUN, Scr, and sNGAL after a higher dosage of LPS intraperitoneal injection. (**B**) Representative figures of renal histopathology; arrows indicate slight tubule enlargement, and red circles refer to mononuclear cell infiltration. (**C**) Concentrations of BUN, Scr, and sNGAL after a lower dosage of LPS intraperitoneal injection. (**D**) Representative figures of renal histopathology from vehicle, 10 μg kg^−1^, and 100 μg kg^−1^ LPS groups. (**E**) Ratio of kidney injury molecule-1 (KIM-1)/creatinine and clusterin/creatinine in urine from vehicle, 10 μg kg^−1^, and 100 μg kg^−1^ LPS groups. (**F**) NGAL protein levels from kidney tissues were determined by western blot. (**G**) Representative figures of immunohistochemistry of NGAL in vehicle, 10 μg kg^−1^, and 20 mg kg^−1^ groups. (**H**) Ly6G protein level in kidney tissues in the 10 μg kg^−1^ group was determined by western blot. (**I**) NGAL protein levels from Human kidney-2 (HK-2) cells stimulated by LPS were determined by western blot. *, *p* < 0.05, **, *p* < 0.01, ***, *p* < 0.001 (*n* = 8 for each subgroup).

LPS i.p. injection to mice is generally used to mimic septic AKI, and the mostly used dosages are 10 mg/kg or 20 mg/kg. As shown in Figure 3A, both 10 mg kg^−1^ and 20 mg kg^−1^ LPS i.p. injections caused significant increases of sNGAL compared to the vehicle control. However, only at 20 mg kg^−1^ dosage did BUN also increase. As shown in Figure 3B, by pathological analysis, both 10 mg/kg and 20 mg/kg dosage caused slight tubular injury with lumen enlargement and mononuclear cell infiltration, which indicated that only sNGAL could discriminate AKI at 10 mg kg^−1^ instead of BUN and Scr.

Besides conventional experimental dosages for septic AKI, very low doses such as 100 μg kg^−1^ and 10 μg kg^−1^ LPS intraperitoneal injection also caused significantly increased sNGAL in mice, and at these doses, both BUN and Scr did not change (Figure 3C). PAS staining also showed that there were no visible pathological injuries under these two lower dosages (Figure 3D). Further, to demonstrate whether LPS at 10 or 100 μg kg^−1^ could cause tubular injury, two other tubular injury biomarkers, clusterin and KIM-1, were tested in the urine. As shown in Figure 3E, KIM-1 and clusterin did not increase in the urine, which provided evidence that tubules were not impaired at these two lower dosages. However, western blot and IHC indicated that the NGAL protein level in kidney tissues significantly increased even under 10 μg kg^−1^ dosage (Figure 3F,G). Aiming to investigate the NGAL source in the kidney, Ly6G, a neutrophil biomarker, was tested by western blot. Results showed that Ly6G significantly increased in kidney tissues in mice under 10 μg kg^−1^ LPS challenge (Figure 3H), suggesting that sNGAL was most likely from the activated neutrophils in vessels in the kidney instead of from injured tubular cells. Immunohistochemistry also demonstrated NGAL was mainly expressed in the tubular area under LPS stimulation, and there was almost no positive staining in glomeruli (Figure 3G). Interestingly, LPS stimulation also caused NGAL protein levels to rise in human tubular HK2 cells, but this was weaker and not dose dependent from 1 ng mL^−1^ to 5000 ng mL^−1^, compared with in vivo results (Figure 3I; *, *p* < 0.05, **, *p* < 0.01).

### 3.3. Serum NGAL Fails to Predict Early Kidney Injury and Progression of Experimental Diabetic Nephropathy

**Figure 4:**
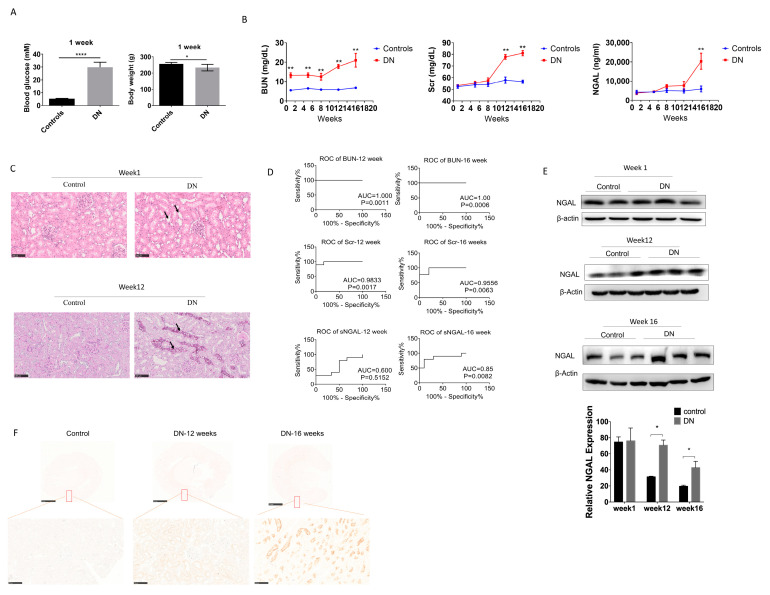
Serum NGAL fails to predict kidney injury in STZ-induced diabetic nephropathy in early stages. (**A**) Comparison of body weight and blood glucose one week after STZ injection. (**B**) BUN, Scr, and sNGAL in experimental diabetic nephropathy (DN) and control groups at different time points. (**C**) Haematoxylin and eosin (H&E) staining of tissue sections at 1 week and 12 weeks after STZ injection. (**D**) Comparison of ROC curves among BUN, Scr, and sNGAL at week 12 and week 16 after STZ injection. (**E**) NGAL protein levels in kidney tissues by western blot at 1 week, 12 weeks, and 16 weeks after STZ injection. (**F**) Representative immunohistochemistry pictures of NGAL at 12 and 16 weeks post glycemia. *, *p* < 0.05, **, *p* < 0.01., *** *p* < 0.0001 (*n* = 8 for each subgroup).

After one week of STZ injection, glycemia was established in SD rats, and body weight began to decrease for each glycemic animal (Figure 4). The concentrations of BUN, Scr, and sNGAL of DN rats and control were measured at 1 week, 5 weeks, 8 weeks, 12 weeks, and 16 weeks, respectively, and are shown in Figure 4B. After one week of STZ injection, BUN had already significantly increased in the DN group compared to the sham control due to the nephrotoxicity caused by STZ, which was confirmed by tubular lumen enlargement and mild necrosis of tubular epithelial cells by H&E staining (Figure 4C). However, sNGAL did not increase in the DN group, and neither did Scr. BUN was significantly higher in the DN group compared to the sham control during the 16 weeks. Scr in the DN group was significantly higher than the sham group from 12 weeks. sNGAL increased significantly until 16 weeks, although it began to slightly increase at 12 weeks (Figure 4B); however, renal interstitium injury was already severe with an abundance of tubular vacuole degenerations at 12 weeks (Figure 4C). ROC curve analysis suggested that from 12 weeks, BUN and Scr had almost 100% AUC, which was better than sNGAL (Figure 4D). The ROC curve suggested sNGAL failed to predict early or middle stages of DN injury, although it had an ROC area 0.85 (*p* = 0.0082) at 16 weeks, but its prediction capability was still lower than BUN and Scr (Figure 4D).

Western blot showed that changes of NGAL protein levels in kidney tissues had a similar trend as that of sNGAL, which began to increase at 12 weeks (Figure 4E), and immunohistochemistry also showed NGAL increased in kidneys in DN rats in a time-dependent manner (Figure 4F). Similar to the AKI models, NGAL staining was mainly located in the tubular area.

### 3.4. Serum NGAL Is More Efficient than BUN and Scr to Discriminate Kidney Impairment in c-BSA-Induced MN Rats Both in Diagnosis and Prognosis

**Figure 5:**
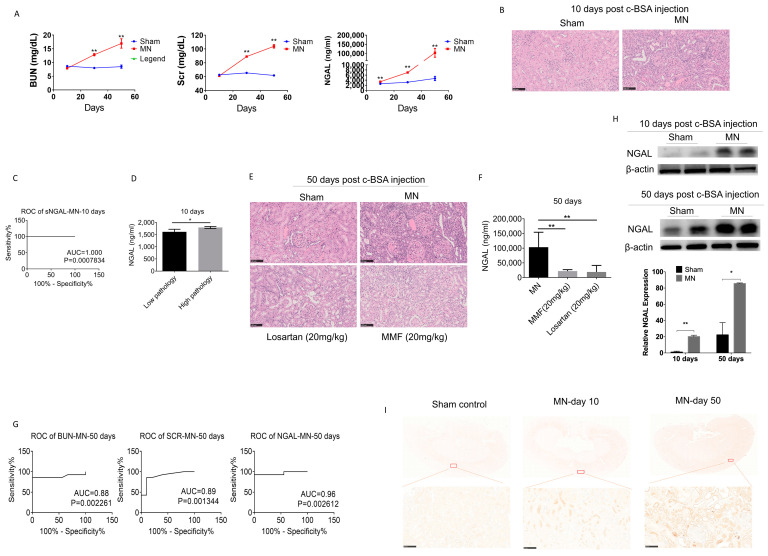
Serum NGAL accurately predicted kidney injury in c-BSA-induced membranous nephropathy. (**A**) Comparisons of BUN, Scr, and sNGAL between the MN model and control after c-BSA injection at three time points. (**B**) Histopathological injuries were evident in the MN model 10 days post c-BSA injection. (**C**) ROC of sNAGL at day 10 after c-BSA injection. (**D**) sNGAL in rats with low-grade pathology was significantly lower than rats with high-grade pathology. (**E**) Histopathological injuries were more evident in the MN model 50 days after c-BSA injection, and MMF and losartan can reduce the renal pathological injuries. (**F**) MMF and losartan treatment remarkably reduced sNGAL in MN rats. (**G**) Comparisons of ROCs of BUN, Scr, and sNGAL between the MN model and control 50 days post BSA injection. (**H**) NGAL protein levels in kidney tissues by western blot at day 10 and day 50 after c-BSA injection. (**I**) Representative immunohistochemistry pictures of NGAL at day 10 and day 50 after c-BSA injection. *, *p* < 0.05, **, *p* < 0.01 (*n* = 8 for each subgroup).

As shown in Figure 1D, at 10, 30, and 50 days after c-BSA i.v. injection, the sera from MN and sham control rats were collected for examination. The concentration curves of BUN, Scr, and sNGAL at different times are shown in Figure 5A, and all these indices increased with time. BUN and Scr did not show significant changes between the two groups after 10 days, but pathological interstitium injuries were observed in the MN group whose major features were infiltration of mononuclear cells and light protein casts (Figure 5B). At this time, sNGAL from MN was significantly higher than the vehicle-treated group, and the predicted ROC was 1 (*p* < 0.001, Figure 5C), which suggests that sNGAL had a distinguished early prediction capability. Moreover, the concentration of sNGAL in high-grade pathology injury was higher than low-grade pathological injury in MN groups 10 days post c-BSA injection (Figure 5D).

After 50 days, c-BSA-induced glomerulonephritis was more serious, with highly increased BUN and Scr as well as abundant protein casts, glomerular hypertrophy, and abundant infiltration of lymphocytes (Figure 5E). MMF and losartan treatments both significantly improved pathological impairments, and they reduced the sNGAL concentration (Figure 5F), which suggests that sNGAL could predict prognosis. At day 50, sNGAL increased 22 times compared to the sham group, and the ROC of sNGAL was larger than that of BUN and Scr, although they also had satisfactory diagnosis abilities (Figure 5G). NGAL protein levels in kidney tissues from the MN model group also increased over time (Figure 5H,I), which was consistent with sNGAL dynamics.

## 4. Discussion

Serum biomarkers are convenient tools to evaluate disease progression and severity, including kidney disease, in experimental models. Although diagnostic values of serum or urine NGAL have already been studied widely, the current investigation still offers novel insight that has not been fully clarified in previous studies. Firstly, the current study thoroughly compared the diagnosis power of sNGAL with BUN and Scr in four experiment KI models, including its performance at different stages. Secondly, we demonstrated that sNGAL was not a good biomarker for early detection in experimental STZ-induced DN (low sensitivity) and LPS-induced AKI (low specificity). Thirdly, sNGAL was not as good a biomarker as BUN and Scr for cisplatin-induced AKI at later stage. Fourthly, sNGAL was a suitable biomarker for immune-mediated experimental glomerulonephritis, not only in early stages but also prognosis at later phases.

Although novel, sensitive biomarkers for KI have emerged quickly, such as IL6, cystatin C, kidney injury molecule-1, NGAL, urine vanin-1, and liver fatty acid-binding protein [18], BUN and Scr are still the most utilized biomarkers to assess kidney injuries in experimental animal models. BUN and Scr are metabolic waste in the human body. Impaired renal function reduces their removal, and this is the principle mechanism of how BUN and Scr can be used as a kidney function biomarker. Although, they cannot be good biomarkers to predict early-stage kidney injury because the kidney has enough reserve capacity [19]. NGAL can be secreted by impaired tubular cells and transferred into the serum and urine, which is mechanism for how NGAL is used as a kidney injury biomarker [4]. Therefore, NGAL is not a direct biomarker of kidney function, not like BUN and Scr. In the current study, we still selected BUN and Scr as references to compare the diagnosis capability with sNGAL.

Experimental models of cisplatin-induced AKI are important to simulate drug-induced nephrotoxicity. Generally, in this animal model, renal impairment reaches its peak 3 days after cisplatin injection [20]. In the current study, at day 1 in the 20 mg/kg dosage group, sNGAL increased significantly compared to BUN and Scr, and the AUC was 100%, which suggests that sNGAL can provide an accurate early diagnosis, and this is consistent with previous studies [21]. However, compared with mouse models of renal ischemia–reperfusion injury, sNGAL increased modestly in cisplatin-induced AKI and was only, on average, 2.65-fold higher 24 h after 20 mg/kg cisplatin administration, while NGAL levels could increase up to 300-fold in blood after 24 h reperfusion in the ischemia–reperfusion injury model [22]. The present study also provides possible evidence that if sNGAL at early stages (within 24 h) increases approximately 40% higher than the average of normal animals, the mouse may die in the following several days, which might be a convincing way to predict lethality in cisplatin-treated mice. Serum NGAL as an independent predictor of mortality has also been reported in a human clinical study, which may serve as a novel outcome-specific marker in intensive care medicine and critical care nephrology to predict mortality in renal replacement therapy [23]. The present study also demonstrates the advantage of sNGAL prediction mainly in early stages. When kidney injury progressed after 72 h, BUN and Scr had higher predictive ROCs than sNGAL, suggesting the sNGAL prediction capability is weaker at later phases. A possible explanation might be that protein concentrations examined by ELISA generally have a high deviation, which reduces the statistical power. In addition, although sNGAL increased under cisplatin stimulation, it seems to be protective for cisplatin-induced AKI because Devarajan et al. reported administration of biologically active recombinant NGAL via the tail vein could significantly ameliorate impaired renal function in cisplatin-induced AKI [24].

LPS i.p. injection mimics sepsis-induced AKI. This experimental model is easier to establish and is usually used to screen compounds against septic AKI in vivo. As shown in Figure 3, even very low doses of LPS injection could cause a higher sNGAL level, but these low dosages cannot produce any visible pathological injuries, and unchanged urine KIM-1 and clusterin also suggest there is no tubular injury under this low LPS dosage. Therefore, as a biomarker, sNGAL has a low specificity and too high a sensitivity to evaluate septic AKI at lower dosage. Although previous studies have demonstrated tubular cells can produce higher NGAL under LPS stimulation [7,25], in the current study, HK2 cells did not produce very high NGAL by LPS stimulation. Remarkably, elevated ly6G in kidney tissue provides evidence that higher renal NGAL mainly results from activated neutrophils in vessels in the kidney under low-dose LPS challenge. These results suggest sNGAL alone is not enough to predict kidney injury when mice are infected with LPS or other bacterial stimuli. However, under high enough toxic dosage, via higher dosage of LPS injection, the NGAL level increases much earlier than BUN and Scr, and its concentration is positively correlated with pathological injuries. In endotoxia conditions, sNGAL is a good biomarker for monitoring renal toxic injuries, which is consistent with other publications.

Diabetic nephropathy is an important complication induced by hyperglycemia, and experimental DN induced by STZ is widely used in drug preclinical development. In the current research, BUN had a satisfactory predictive capability based on the ROC curve during the entire period, from the early stage to middle–later stages, but a higher BUN in the early phase might be due to acute kidney injury caused STZ nephrotoxicity instead of chronic glomerular endothelial cell injury or tubular epithelial cell impairment caused by hyperglycemia [26]. Scr has a better predictive capability in the middle–late stages (12 weeks or later post glycemia), which is more reflective of real DN progression. However, in the present study, sNGAL did not increase significantly until 16 weeks post glycemia, but even at the 12-week time point renal interstitial pathological injury was already severe; therefore, sNGAL was not advantageous in experimental DN diagnosis in the current study compared with Scr at the middle stage. According to western blot and immunohistochemistry, after 12 weeks of hyperglycemia, NGAL protein levels increased modestly in the kidney tubular area but not enough to cause an obvious increase in blood. We also speculate that most NGAL proteins produced by impaired tubular cells at this stage are not reabsorbed into the blood, but they enter into the tubular lumen and finally into the urine. This is a possible reason why most NGAL examinations in experimental and clinical DN are tested using urine samples [27,28,29,30], and most studies demonstrate that the increase in urinary NGAL is significant compared to the blood NGAL concentration [31,32]. After 16 weeks of glycemia, NGAL was extraordinarily produced by impaired tubular cells, which was demonstrated by western blot and immunohistochemistry, and we can find that NGAL was significantly increased in blood and also had a better ROC diagnosis power. Our studies suggest that sNGAL is not a good biomarker to predict kidney injuries in experimental diabetic nephropathy at early and middle stages, and we also obtained similar results using Wistar rats (data not shown). Individual differences in animals between different batches may affect the time when NGAL increases, but the most likely reason we postulate is that NGAL production in rat tubular cells may not be sensitive to glycemia-induced tubular injury, which is different from drug-induced or immune-mediated injuries, and these injuries may cause more pronounced NGAL production. The role of NGAL in DN is still not clear, and its biofunction in DN initiation and progression deserves in-depth study.

Intravenous c-BSA-induced MN is a convenient experimental immune-mediated glomerulonephritis animal model. Elevated urinary NGAL levels have been reported in immune-mediated nephropathy, including membranous nephropathy and primary focal segmental glomerulosclerosis [33,34], but investigation of sNGAL is rare. Previous studies using anti-glomerular basement membrane (GBM) glomerulonephritis rats or mice both suggest sNGAL increases in early stages, and they also prove sNGAL is positively correlated with kidney pathological injuries, suggesting it might be a good biomarker for experimental immune-mediated glomerulonephritis [35,36]. In the current study, sNGAL provides an accurate early diagnosis power compared to BUN and Scr. Meanwhile, the sNGAL concentration is positively correlated with pathological injuries, which confirms that sNGAL is a reliable early biomarker for immune-mediated experimental MN. In the later stages of MN, BUN and Scr also increased, but the AUC of the ROC curve of sNGAL was better than that of BUN and Scr. After MMF and losartan treatment, sNGAL significantly decreased, which suggests that sNGAL is also a satisfactory prognosis biomarker. Several publications demonstrate that podocytes could express NGAL under inflammation, which is another possible source of sNGAL [7]; however, IHC results from the present study suggest most staining of NGAL is still in the tubular area, and not glomeruli, which is consistent with the results from anti-GBM glomerulonephritis in rodents where NGAL is expressed in tubular interstitium. This research also demonstrates that NGAL overexpression can aggravate renal injuries and is harmful for immune-mediated glomerulonephritis, not like in AKI which shows a renal protective effect [36].

The limitations of the current study are that we only focused on the diagnosis and prognosis of sNGAL, a functional study of NGAL in kidney disease is lacking, and also only four experimental KI models were investigated and cannot represent other KI models.

## 5. Conclusions

In summary, sNGAL could provide an accurate early diagnosis of drug-induced kidney injury, but it is not superior in later phases compared to BUN and Scr. For septic AKI, more attention must be paid due to its low specificity. The early diagnostic value of sNGAL in diabetic nephropathy has not been satisfied. Serum NGAL is valuable for experimental immune-mediated glomerulonephritis, both for early diagnosis and prognosis.
